# Fabrication of gallium nitride and nitrogen doped single layer graphene hybrid heterostructures for high performance photodetectors

**DOI:** 10.1038/s41598-020-71514-9

**Published:** 2020-09-02

**Authors:** Sanjay Sankaranarayanan, Prabakaran Kandasamy, Ramesh Raju, Baskar Krishnan

**Affiliations:** 1 Department of Electronics and Communication Engineering, Koneru Lakshmaiah Education Foundation, Hyderabad, 500 075 Telangana India; 2 Crystal Growth Centre, Anna University, Tamil Nadu, Chennai, 600 025 India; 3 Key Laboratory for Micro-Nano Physics and Technology of Hunan Province, College of Materials Science and Engineering, Hunan University, Changsha, 410 082 People’s Republic of China; 4 Department of Electronics and Nanoengineering, Aalto University, P O BOX 13500, 00076 Espoo, Finland

**Keywords:** Engineering, Materials science, Nanoscience and technology, Physics

## Abstract

Gallium nitride (GaN) was epitaxially grown on nitrogen doped single layer graphene (N-SLG) substrates using chemical vapour deposition (CVD) technique. The results obtained using x-ray diffractometer (XRD) revealed the hexagonal crystal structure of GaN. Photoluminescence (PL) spectroscopy, energy dispersive x-ray (EDX) spectroscopy and x-ray photoelectron (XPS) spectroscopy revealed traces of oxygen, carbon and nitrogen occurring either as contamination or as an effect of doping during the GaN growth process. In addition, PL revealed a weak yellow luminescence peak in all the samples due to the presence of N-SLG. From the obtained results it was evident that, presence of N-SLG underneath GaN helped in improving the material properties. It was seen from the current–voltage (I–V) response that the barrier height estimated is in good agreement with the Schottky–Mott model, while the ideality factor is close to unity, emphasizing that there are no surface and interface related inhomogeneity in the samples. The photodetector fabricated with this material exhibit high device performances in terms of carrier mobility, sensitivity, responsivity and detectivity. The hall measurement values clearly portray that, the GaN thus grown possess high electron contents which was beneficial in attaining extraordinary device performance.

## Introduction

The fascinating properties of gallium nitride (GaN) such as wide direct band gap nature, ability to tune the band gap, high breakdown voltage, carrier mobility and chemical stability, make GaN a widely explored semiconductor material. The ability to operate at high power, high frequency and tolerance towards harsh environments is also a reason for preferring GaN^1–8^. Conventionally, GaN is epitaxially grown using metal organic chemical vapour deposition (MOCVD), molecular beam epitaxy (MBE), hydride vapour phase vapour epitaxy (HVPE) and chemical vapour deposition (CVD) techniques^9–12^.

It is worth to note that GaN is still being epitaxially grown on foreign substrates such as silicon (Si), sapphire (Al_2_O_3_) and silicon carbide (SiC). Sapphire is conventionally preferred as the substrate material due to its hexagonal crystal structure, availability in high crystalline quality and large area. But, due to the thermal and lattice variations between sapphire substrates and GaN, the aforesaid material properties of GaN cannot be achieved effectively. Also, poor thermal conductivity of sapphire restricts the usage of GaN in high power and optoelectronic devices^13–23^. To overcome these deficiencies, it is beneficial to utilize graphene as an intermediate layer for the growth of GaN.

Graphene, a two-dimensional (2D) material with a planar honeycomb like configuration of sp^2^ hybridized carbon atoms, has attracted enormous interest for use in various optical and electronic device applications due to its unique material properties such as high optical transparency, thermal and electrical conductivity and mechanical properties^24,25^. Numerous techniques like mechanical cleavage from bulk graphite, CVD, reduction of graphene oxides (GO) from bulk graphite, deposition of ultra-thin graphite from SiC decomposition and liquid phase exfoliation, are being employed for the preparation of graphene^26–30^. With current technologies, synthesis of high-quality graphene and its transfer onto desired substrates is achievable with a prospect of graphene becoming a cheap alternative available in abundance^25^. By reducing the dimension of graphene into one-dimensional (1D) graphene nanoribbons (GNR) and zero-dimensional (0D) graphene quantum dots (GQD), the material properties of graphene can be tuned to a greater extent making it a suitable material for electrocatalysis, sensors, energy conversion and storage applications^31^. Apart from control of dimension, chemical doping is also an important aspect to improve the properties of graphene. To be specific, graphene is chemically doped by substituting nitrogen (N) atoms into the hexagonal crystal lattice to improve its electronic properties^31^.

Despite these merits, it is to be noted that graphene does not favour nucleation sites for the growth of GaN due to absence of dangling bonds. Consequently, epitaxial growth of GaN on graphene leads to the formation of three dimensional (3D) GaN clusters, resulting in poor growth quality of GaN films on graphene, which is significantly worse than that of GaN films grown on a conventional SiC or sapphire substrate. Therefore, to favour nucleation, either a thin layer of aluminium nitride (AlN) or zinc oxide (ZnO) nanowalls is to be deposited on graphene as an intermediate layer^32^. Also, substitutional doping of nitrogen atoms into graphene, tend to distort its crystal structure by producing defects such as vacancies, bonding disorders and non-cyclized structures. The small covalent radius and higher electronegativity of nitrogen atom also significantly influence the structural and electronic properties of graphene^31^.

In the present work, growth of GaN was carried out using CVD technique on a nitrogen doped single layer graphene (N-SLG) substrate. Uniqueness of this study is the growth of better quality GaN without deposition of an intermediate layer on N-SLG. Fabrication of GaN grown on N-SLG substrates into a metal–semiconductor-metal (MSM) based highly sensitive photodetectors, to the best of our knowledge, have not been explored earlier. This manuscript revolves around the analysis of substitutional doping and contaminations occurring during the growth process for the usage of GaN in next-generation semiconductor devices.

## Results and discussion

### Structural analysis

**Figure 1:**
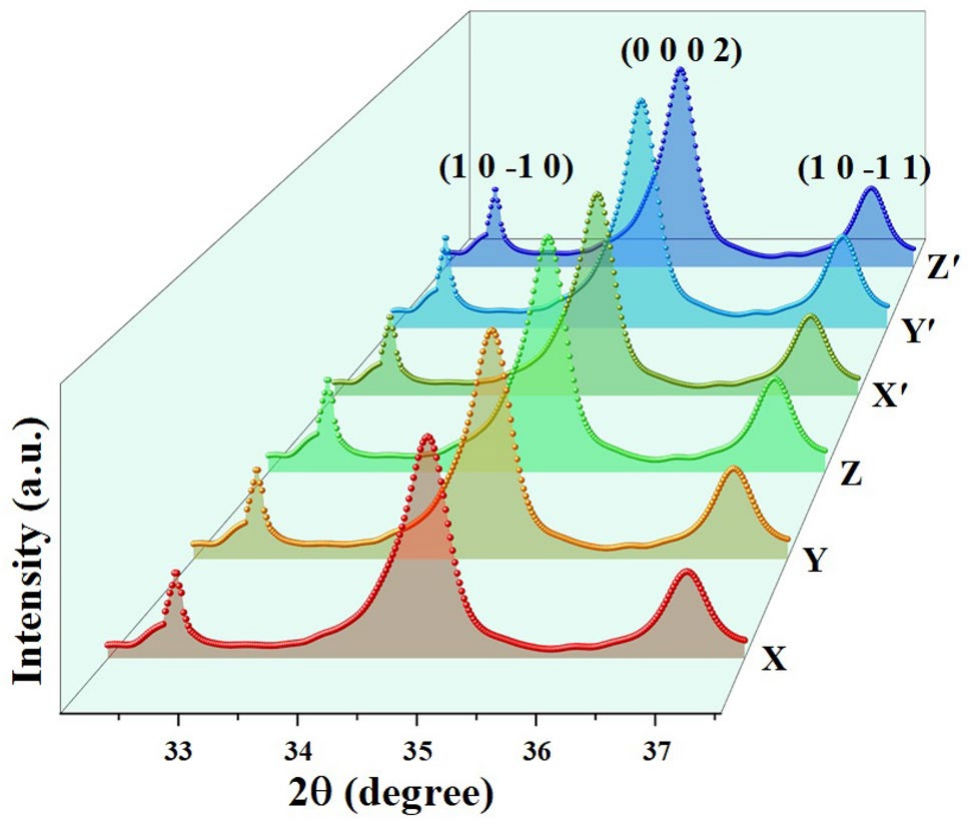
XRD pattern of GaN grown on N-SLG substrates by varying the growth time (X, Y and Z) and precursor-to-substrate distance (Xʹ, Yʹ and Zʹ).

The structural information of GaN grown on N-SLG substrates by varying the experimental conditions were obtained using XRD as shown in Fig. 1. Dominant diffraction peaks corresponding to GaN were obtained at $\left( {1 0 - 1 0} \right)$, $\left( {0 0 0 2} \right)$ and $\left( {1 0 - 1 1} \right)$ planes. The indexed peaks revealed that GaN exhibit hexagonal crystal structure and match well with the JCPDS data. The peak dominant at $\left( {0 0 0 2} \right)$ plane confirm that the growth of GaN is along c-axis. These amplified peaks exemplify the crystalline quality of the GaN. It is worth to note that the full width at half maximum (FWHM) value estimated for the GaN epitaxially grown on N-SLG substrates at $\left( {0 0 0 2} \right)$ plane is around 0.75°, indicating good growth along c-axis. It can be seen from literature that Chung et. al attained almost similar FWHM (0.81°) for GaN films grown on CVD graphene^33^. Similarly, Yadav et. al and Sanjay et. al achieved FWHM of around 3–6° for GaN films grown on ZnO and few layer graphene (FLG) respectively^34,35^. Wong et. al obtained a polytype growth for gallium-based compound materials grown on multi-layer graphene (MLG)^36^. Hence, it is evident that, irrespective of number of layers (SLG / MLG / FLG), presence of graphene as an intermediate layer has a critical role in enhancing the crystalline quality of GaN.

### Surface morphology analysis

**Figure 2:**
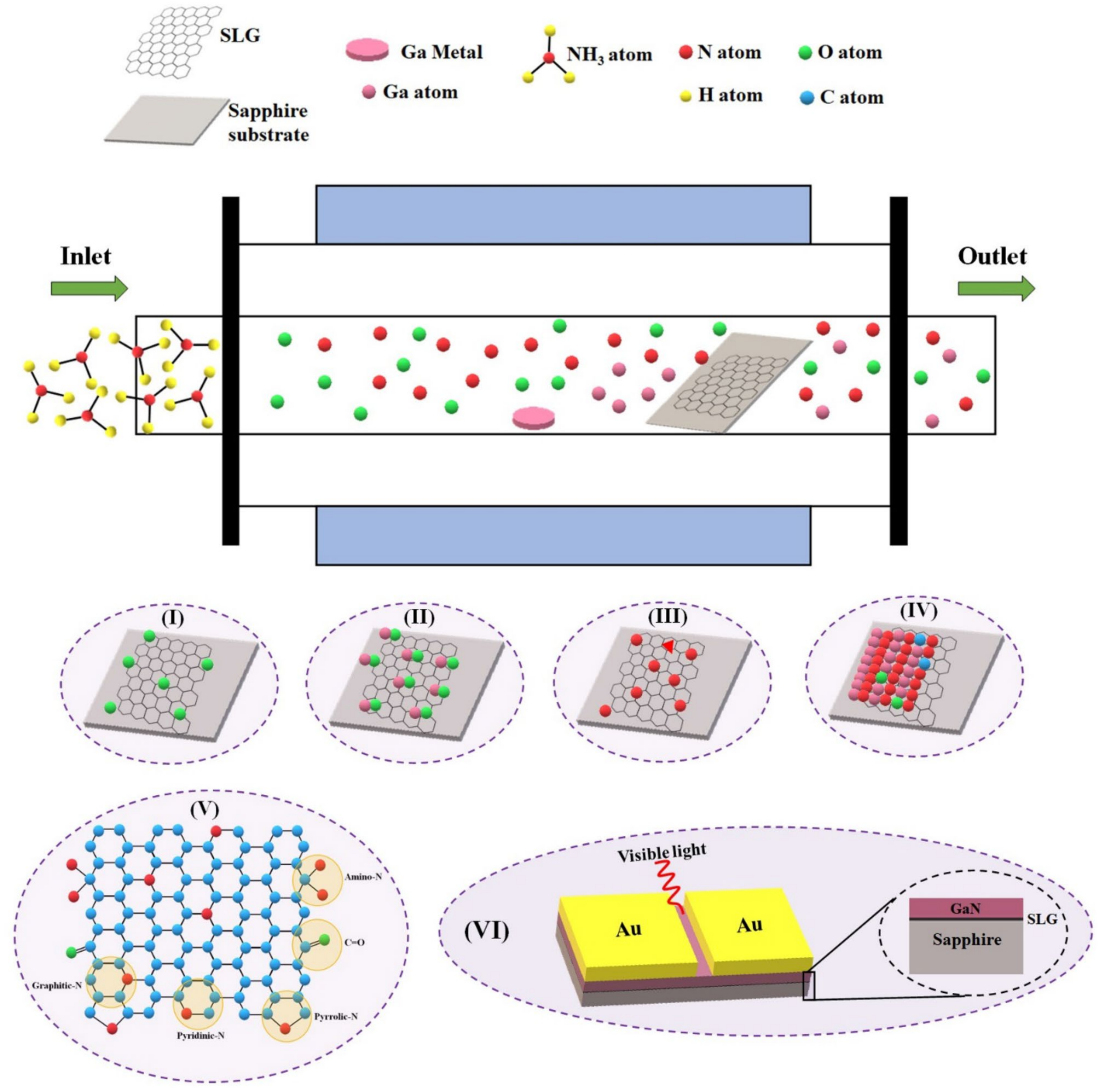
Schematic representation of GaN growth process. (I) attachment of residual oxygen on SLG, (II) formation of GaO_x_ complex, (III) doping of nitrogen atoms into the graphene lattice, (IV) growth of GaN on N-SLG, (V) schematic of graphene as a combination of the above process, (VI) fabrication of metal–semiconductor-metal based photodetectors.

The schematic portraying GaN growth process on N-SLG substrate in a CVD reactor is shown in Fig. 2 which gives a clear picturization about the growth process.

**Figure 3:**
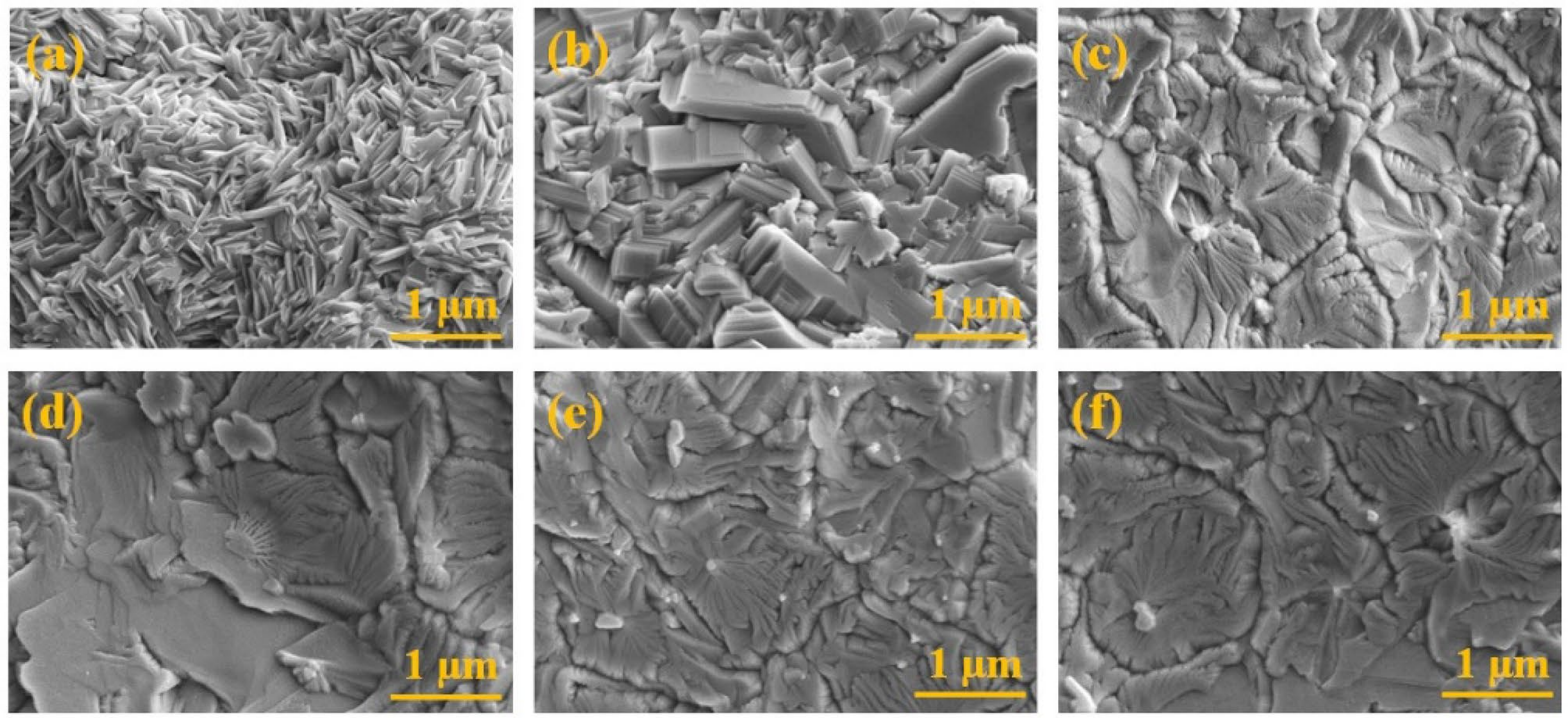
SEM images representing: (**a**) sample X, (**b**) Y, (**c**) Z, (**d**) Xʹ, (**e**) Yʹ and (**f**) Zʹ.

The surface morphology of GaN grown on N-SLG substrates were obtained using SEM as shown in Fig. 3. The growth of GaN was carried out in two different categories by varying the: (a) growth time as 60, 90 and 120 min, referred as sample X, Y and Z respectively and (b) precursor-to-substrate distance as 3, 5 and 7 cm, referred as sample Xʹ, Yʹ and Zʹ respectively.

In respect of growth time of 60 min (Fig. 3a), densely packed GaN flakes were observed on the N-SLG substrates. It may be noted that, initially sparse nuclei islands will be formed during the nucleation stage. These islands get attached to the dangling bonds present in graphene which occurred as an effect of residual oxygen (O) present within the CVD reactor. The oxygen atoms tend to taint the gallium metal (used as gallium precursor) by forming gallium oxide (GaO_x_) complex. This produces GaO_x_ droplets on the N-SLG substrates during the initial growth process. However, as the growth proceeds, the oxygen atoms are replaced by nitrogen atoms^37^. As an effect of growth temperature, re-crystallization of islands is favoured, leading to the formation of dense GaN flakes like morphology^38^. When the growth time was increased to 90 min (Fig. 3b), the nucleation rate increased, resulting in the formation of more dense nucleation layers by adsorbing more and more gallium and nitrogen adatoms. These dense nucleation layers provide ample amount of adsorption sites on the surface and favours more re-crystallization leading to the formation of large nuclei islands. When the growth time was further increased to 120 min (Fig. 3c), the 3D islands became larger, resulting in coalescence of islands. This tends to transform the GaN from 3D islands to quasi 2D films^39^. However, in respect of precursor-to-substrate distance, only marginal variations in the morphology of GaN films were noticed (Fig. 3(d-f)). This is because, as reported in the literature, gallium vapour concentration decreases with increase in the precursor-to-substrate distance, resulting in shortage or improper supply of gallium species. The results obtained are in good agreement with the literature^40^.

**Figure 4:**
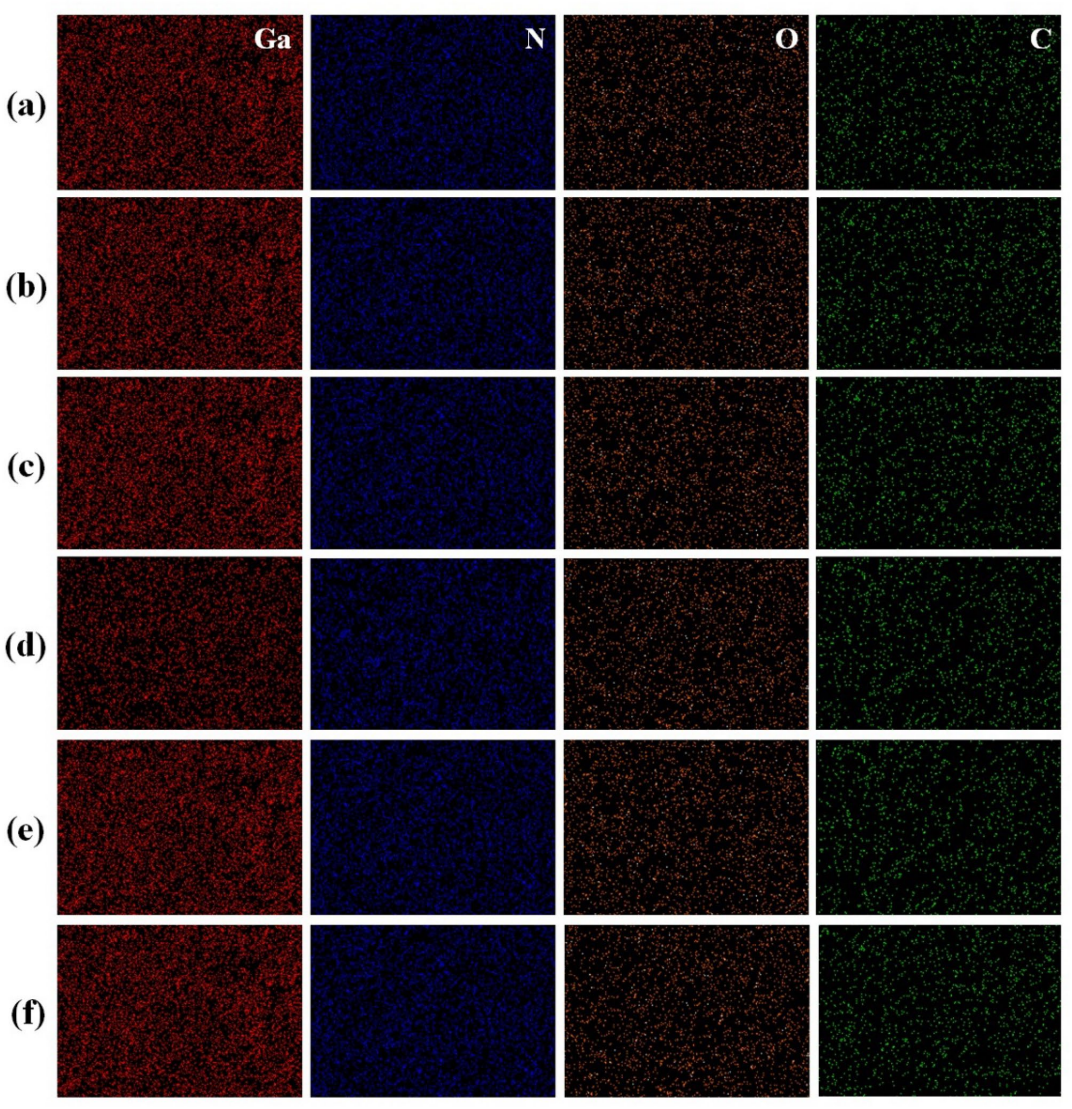
EDX maps revealing gallium (red colour), nitride (blue colour), oxygen (orange colour) and carbon (green colour) contents in samples (**a**) X, (**b**) Y, (**c**) Z, (**d**) Xʹ, (**e**) Yʹ and (**f**) Zʹ respectively.

The energy dispersive x-ray (EDX) spectroscopy maps obtained using SEM were utilized to analyse the material composition of GaN grown on N-SLG surface as shown in Fig. 4. It is seen from the Figure that in respect of all the samples, carbon (C) and oxygen traces were observed in addition to gallium and nitrogen. The carbon and oxygen traces can be attributed either as extrinsic contamination or contamination induced during the GaN growth process. The effect of oxygen contamination occurring during the growth process has been discussed already. The carbon contamination can be due to the decomposition of NH_3_ into nitrogen atoms as an effect of growth temperature^41^. This process occurs simultaneously along with the oxygen contamination process and tends to dope nitrogen atoms into the graphene due to the presence of dangling bonds. Therefore, nitrogen doping will benefit in not only replacing the oxygen atoms in the GaO_x_ complex with nitrogen, but also in improving the material properties of graphene. According to literature, nitrogen atoms gets doped into graphene either as graphitic-N or pyridinic-N or pyrrolic-N configuration^31^. The process of doping tends to improve the electronic properties in graphene. Another advantage in doping is that, when nitrogen atoms get incorporated into the graphene’s honeycomb lattice, it tends to distort the crystal structure by creating disorders^42^. This is because, the lattice difference between GaN and graphene is around 29.6%. The high strain energy between them lead to the structural deformation in graphene thereby, making it defective. Therefore, doping SLG with nitrogen is also expected to improve nucleation rate during the growth of GaN. Similar phenomena were reported by Y. Gohda et al. using density functional theory (DFT) calculations. He illustrated that GaN grown on graphene tend to break the sp^2^ C–C bonds partially by forming C-N–C bonding to overcome the in-plane strain in the graphene lattice^43^. Also, M. Gruart et al.^44^ observed the formation of rough and imperfectly coalesced GaN clusters along defective regions in the graphene, which is consistent with the results obtained by us.

Therefore, it is concluded that, the above three processes such as: formation of GaO_x_ complex, nitrogen doping into the honeycomb lattice of graphene and defect formation due to lattice difference between graphene and GaN, tend to create carbon, nitrogen and oxygen related point defects such as vacancies, interstitials and substitutional impurities and structural distortions in GaN. However, it is also to be noted that these actions have directly or indirectly contributed towards the nucleation process for the growth of GaN and also helped in improving the material properties.

### Surface composition analysis

**Figure 5:**
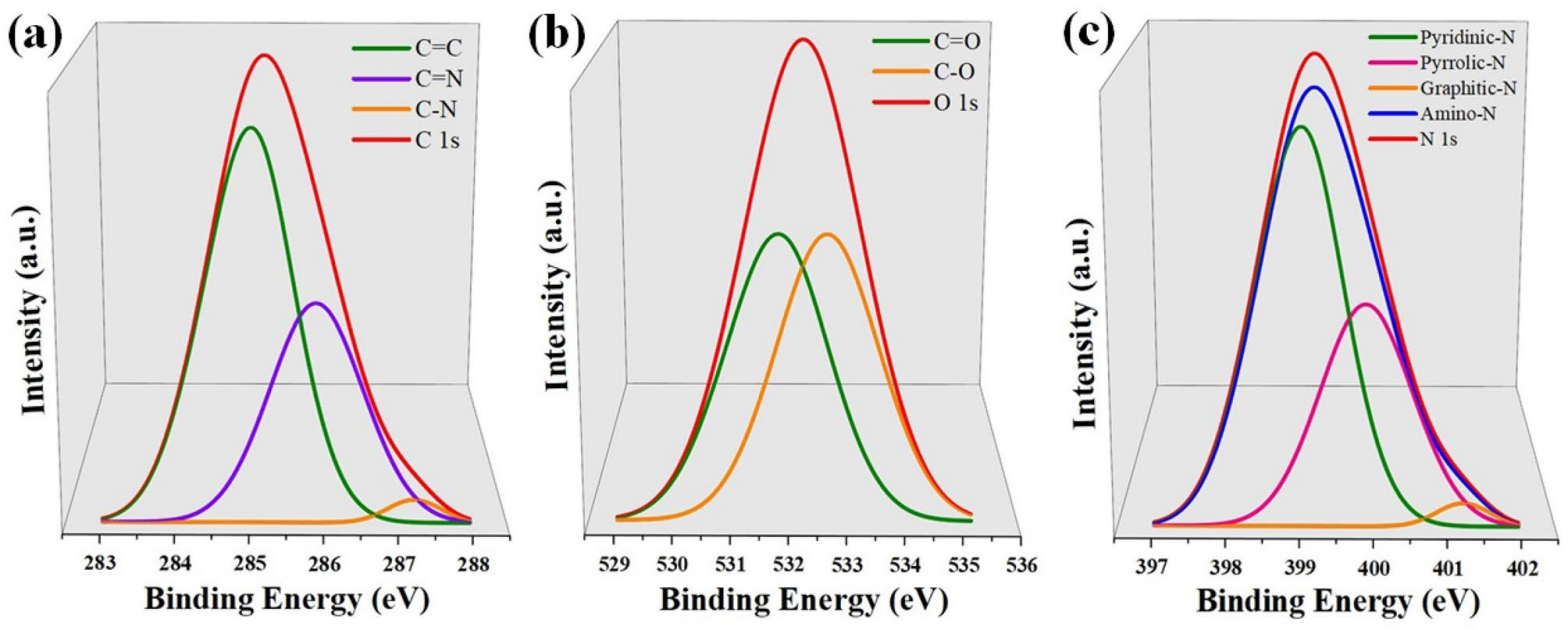
Individual XPS spectra of GaN representing: (**a**) carbon, (**b**) oxygen and (**c**) nitrogen.

Incorporation of nitrogen, carbon and oxygen atoms into graphene cannot be justified based only on SEM and EDX results. Hence, the samples were characterized using XPS to determine the surface composition (Fig. 5). The obtained peaks corresponding to nitrogen, carbon and oxygen were deconvoluted to obtain their functionalities. The carbon peak was deconvoluted into three subpeaks associated to C = C (${\text{sp}}^{2}$ hybridized), C = N (${\text{sp}}^{2}$ hybridized) and C–N (${\text{sp}}^{3}$ hybridized) located at 284.5, 285.8 and 287.5 eV respectively (Fig. 5a). From the carbon spectrum, no peak was detected at 289 eV, emphasizing absence of physisorbed oxygen in the graphene^31,45^. This concludes that, a substantial quantum of oxygen atoms were replaced with nitrogen atoms either during the growth of GaN or when nitrogen atoms are getting doped into graphene as substitutional impurities, leading to the formation of C–N bonds.

The deconvolution of oxygen spectrum revealed two subpeaks at 531.4 and 532.4 associated to C = O and C–O respectively, which could be attributed to the presence of different oxygen functionalities (Fig. 5b)^31,42^. The presence of oxygen functionalities could be due to the effect of GaOx complex formation during the initial nucleation stages of GaN.

Nitrogen peak was deconvoluted into four subpeaks associated to pyridinic-N, pyrrolic-N, graphitic-N and amino-N located at 398.4, 400.1, 401.4 and 399.1 eV respectively (Fig. 5c). It is to be noted that, pyridinic-N is ${\text{sp}}^{2}$ hybridized, where nitrogen atoms specifically get attached to two carbon atoms at the edges or defects of graphene. Whereas, pyrrolic type is ${\text{sp}}^{3}$ hybridized, in which nitrogen atoms binds into the five-membered carbon ring. Similar to pyridinic-N, graphitic-N is also ${\text{sp}}^{2}$ hybridized, where nitrogen atoms substitute for carbon atoms within the hexagonal ring^31,42,45,46^. It is to be noted that, the amount of nitrogen atoms getting doped into SLG will be very low. This is due to high temperature utilized for the growth of GaN which breaks most of the C-N bonds formed^42,47^. Thus, the elemental traces of nitrogen getting doped into the SLG were observed and confirmed.

### Luminescence characteristics

**Figure 6:**
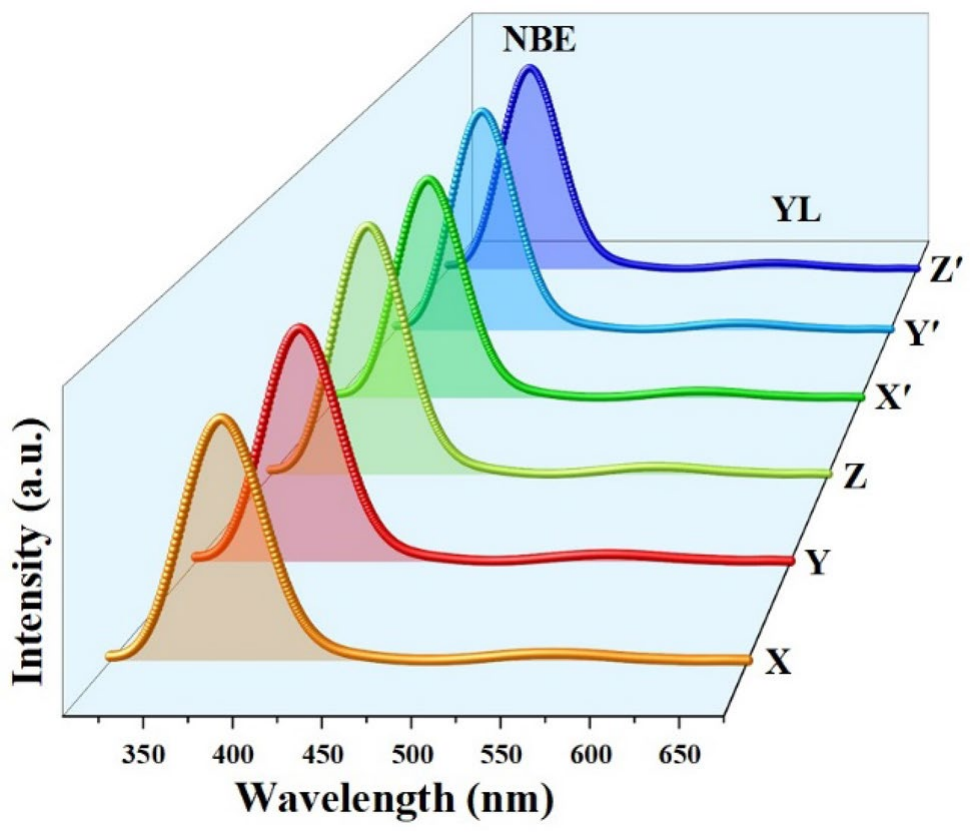
PL spectra of GaN grown on N-SLG substrates by varying the growth time (X, Y and Z) and precursor-to-substrate distance (Xʹ, Yʹ and Zʹ).

The luminescence characteristics of GaN samples grown on SLG substrates by altering the time of growth and precursor-to-substrate distance are shown in Fig. 6. It can be seen that, a strong emission peak, known as near band edge (NBE) was observed at around 3.45 eV. The NBE emission is attributed to the radiative recombination of electron from excited state to the ground state^48,49^.

In addition to NBE, a weak, yet a broad emission peak known as yellow luminescence (YL) was observed at around 2.2 eV. It is to be noted that, irrespective of any growth technique and / or experimental conditions, the presence of YL can be observed usually in undoped and n-doped GaN. The YL is attributed to the radiative transition from a shallow donor to a deep acceptor or due to point defects such as gallium vacancies $\left( {{\text{V}}_{{{\text{Ga}}}} } \right)$, oxygen substituting gallium $\left( {{\text{O}}_{{{\text{Ga}}}} } \right)$ or oxygen substituting nitrogen $\left( {{\text{O}}_{{\text{N}}} } \right)$^48–53^ and surface defects such as excitons bound to shallow or structural defects, stacking faults and screw dislocations^54–62^.

It is worth to note that, the presence of N-SLG underneath GaN has effectively quenched the YL by a greater extent when compared with the YL in GaN without interlayer. However, it was inferred that, the presence of SLG is also a reason for the YL observed in GaN. This is because, carbon atoms get doped into GaN in various forms such as carbon substituting nitrogen $\left( {{\text{C}}_{{\text{N}}} } \right)$, carbon substituting gallium $\left( {{\text{C}}_{{{\text{Ga}}}} } \right)$, forming carbon – oxygen complex substituting nitrogen $\left( {{\text{C}}_{{\text{N}}} - {\text{O}}_{{\text{N}}} } \right)$ and complex between gallium vacancy and carbon $\left( {{\text{C}}_{{\text{N}}} - {\text{V}}_{{{\text{Ga}}}} } \right)$. The YL is a transition either from a shallow donor to a deep acceptor or from a deep donor to a shallow acceptor, which is in good agreement with the literature^48–53^. Therefore, carbon acts as a parasite degrading the material quality of GaN.

Comparing with literature, M. Heilmann et al.^25^ attained the ratio of intensities between YL and NBE $\left( {{\text{I}}_{{{\text{YL}}}} /{\text{I}}_{{{\text{NBE}}}} } \right)$ value at around 2 for GaN micro- and nano-rods grown on SLG substrates, emphasizing the fact that YL dominates. Similarly, H. Yang et al.^63^ attained the value of ${\text{I}}_{{{\text{YL}}}} /{\text{I}}_{{{\text{NBE}}}}$ as 5 for GaN grown on reduced graphene oxide (rGO) sheets. However, S.J. Chae et al.^64^ did not observe YL peak but, observed the donor–acceptor pair (DAP) transitions for GaN grown on FLG, where DAP is attributed to the recombination of electron bound to a shallow donor with a diffuse hole bound to a shallow acceptor. By comparing the above results, S. Sanjay et al.^35^ attained the value of ${\text{I}}_{{{\text{YL}}}} /{\text{I}}_{{{\text{NBE}}}}$ at around 0.4 for GaN pyramids grown on FLG substrates, resulting in the suppression of YL peak. In the present scenario, from the Figure, the value of ${\text{I}}_{{{\text{YL}}}} /{\text{I}}_{{{\text{NBE}}}}$ is estimated at around 0.05 – 0.15, confirming that carbon also contributes to the YL in GaN but, to a very small extent. This can be correlated to the defects in the graphene occurring during the GaN growth process. Thus, the PL results obtained corroborate with SEM data.

From the Figure, it was observed that, irrespective of variations in either growth time or precursor-to-substrate distance, there were no significant change in the peak position of NBE. However, slight variations were observed in relation to the peak intensities of the samples. On comparing the results obtained by S. Sanjay et al.^35^, it is evident that YL increases with number of layers.

### Electrical and optical studies

**Figure 7:**
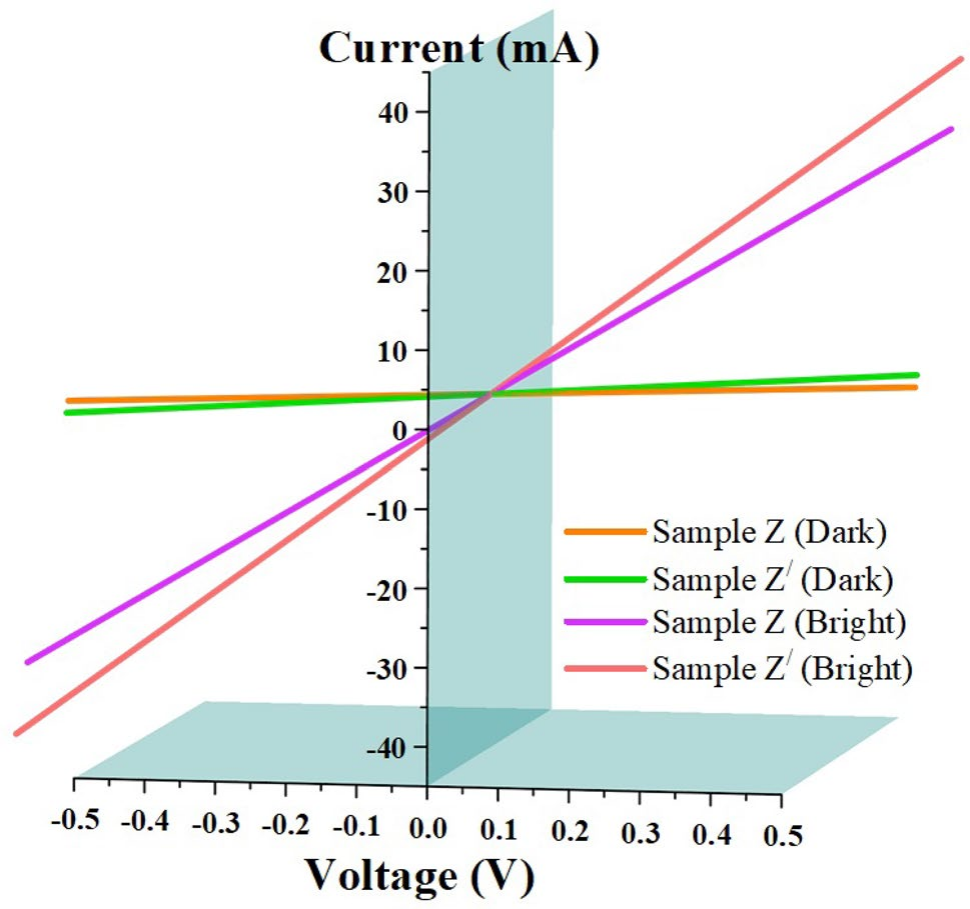
I–V response in respect of samples Z and Zʹ tested under dark and bright conditions.

Based on the surface morphologies obtained using SEM, the samples Z and Zʹ were preferred for carrying out the current–voltage (I–V) characteristics (Fig. 7). This is because, sample Z represents the transformation in morphology of GaN from 3 to 2D. Also, sample Zʹ portray better surface morphology in comparison to all other samples. Therefore, in the present study, only these two samples were preferred for obtaining the electrical performance.

The electrical response was measured at an applied bias voltage of 0.5 V, both in the presence (bright) and absence (dark) of light as shown in Fig. 7. It was observed from the Figure that the obtained I–V curves were linear and symmetrical, confirming that the carrier density in GaN is high. Since the electron affinity of GaN $\left( {{\upchi }_{{{\text{GaN}}}} = 4.2\;{\text{eV}}} \right)$ is less than the work function of Au $\left( {\phi_{{\text{n}}} = 5\;{\text{eV}}} \right)$, the contacts formed will be of Schottky type^35^.

In the case of dark condition, the charge transfer across the metal–semiconductor (MS) interface is to be considered, which is obtained by calculating the barrier height $\left( {\phi_{{\text{B}}} } \right)$ and ideality factor $\left( {\upeta } \right)$ between the MS junctions. The expression for calculating $\phi_{{\text{B}}}$ and ${\upeta }$ can be given as^65^

1 $$ \phi_{{\text{B}}} = \left( {{\raise0.7ex\hbox{${{\text{KT}}}$} \!\mathord{\left/ {\vphantom {{{\text{KT}}} {\text{q}}}}\right.\kern-\nulldelimiterspace} \!\lower0.7ex\hbox{${\text{q}}$}}} \right){\ln}\left( {{\raise0.7ex\hbox{${{\text{AA}}^{*} {\text{T}}^{2} }$} \!\mathord{\left/ {\vphantom {{{\text{AA}}^{*} {\text{T}}^{2} } {{\text{I}}_{{\text{s}}} }}}\right.\kern-\nulldelimiterspace} \!\lower0.7ex\hbox{${{\text{I}}_{{\text{s}}} }$}}} \right) $$

2 $$ {\upeta } = \left( {{\raise0.7ex\hbox{${\text{q}}$} \!\mathord{\left/ {\vphantom {{\text{q}} {{\text{KT}}}}}\right.\kern-\nulldelimiterspace} \!\lower0.7ex\hbox{${{\text{KT}}}$}}} \right) \cdot \left( {{\raise0.7ex\hbox{${\partial {\text{V}}}$} \!\mathord{\left/ {\vphantom {{\partial {\text{V}}} {\partial (\ln {\text{I}})}}}\right.\kern-\nulldelimiterspace} \!\lower0.7ex\hbox{${\partial (\ln {\text{I}})}$}}} \right) $$

where ${\text{A}}$ is the area of Schottky contact, ${\text{q}}$ is the electronic charge, ${\text{V}}$ is the applied voltage, ${\text{T}}$ is the temperature in Kelvin, ${\text{K}}$ is the Boltzmann constant $\left( {1.38{ } \times { }10^{ - 23} \;{\text{m}}^{2} \;{\text{kg}}\;{\text{s}}^{ - 2} \;{\text{K}}^{ - 1} } \right)$, ${\text{A}}^{*}$ is the Richardson’s constant ($26.8\;{\text{A}}\;{\text{cm}}^{ - 2} \;{\text{K}}^{ - 2}$ for GaN) and ${\text{I}}_{{\text{s}}}$ the reverse saturation current derived from thermionic emission model as shown below^65^

3 $$ {\text{I}} = {\text{I}}_{s} \left[ {{\text{e}}\left( {{\raise0.7ex\hbox{${{\text{qV}}}$} \!\mathord{\left/ {\vphantom {{{\text{qV}}} {\upeta {\text{KT}}}}}\right.\kern-\nulldelimiterspace} \!\lower0.7ex\hbox{${\upeta {\text{KT}}}$}}} \right) - 1} \right] $$

4 $$ {\text{I}}_{{\text{s}}} = {\text{AA}}^{*} {\text{T}}^{2} {\exp}\left( {{\raise0.7ex\hbox{${ - {\text{q}}\phi_{{\text{B}}} }$} \!\mathord{\left/ {\vphantom {{ - {\text{q}}\phi_{{\text{B}}} } {{\text{KT}}}}}\right.\kern-\nulldelimiterspace} \!\lower0.7ex\hbox{${{\text{KT}}}$}}} \right) $$

The barrier height and ideality factor in respect of sample Z and Zʹ were estimated as 0.81 – 0.83 eV and 0.90 – 0.91 respectively. It was observed that, the barrier height and ideality factor values ascertained are in good agreement with the theoretical limit, confirming the MS interface is free from barrier inhomogeneities and interfacial surface states. From the Figure, the dark current $\left( {{\text{I}}_{{{\text{dark}}}} } \right)$ for sample Z and Zʹ were determined as 0.2 and 0.4 A respectively. Even though, both the samples exhibit high values of dark current, the value exhibited by sample Z is less than that of sample Zʹ, which could be attributed to a poor interface in sample Z (where the morphology transforms from 3 to 2D). Irrespective of type of morphologies, the presence of SLG underneath GaN benefited in improving the dark current.

Similarly, under bright condition, the photo conversion efficiency across the MS interface is to be considered, which is obtained by estimating the photo current $\left( {{\text{I}}_{{{\text{photo}}}} } \right)$, sensitivity $\left( {\text{S}} \right)$, responsivity $\left( {\text{R}} \right)$ and detectivity $\left( {\text{D}} \right)$ using the following Eqs. ^66–68^.

5 $$ {\text{I}}_{{{\text{photo}}}} \left( {\text{A}} \right) = {\text{I}}_{{\max}} - {\text{I}}_{{{\text{dark}}}} $$

6 $$ {\text{S}} = \left( {{\raise0.7ex\hbox{${{\text{I}}_{{{\text{photo}}}} }$} \!\mathord{\left/ {\vphantom {{{\text{I}}_{{{\text{photo}}}} } {{\text{I}}_{{{\text{dark}}}} }}}\right.\kern-\nulldelimiterspace} \!\lower0.7ex\hbox{${{\text{I}}_{{{\text{dark}}}} }$}} } \right) \times { }100{\text{\% }} $$

7 $$ {\text{R }}\left( {{\text{A}}/{\text{W}}^{ - 1} } \right) = {\raise0.7ex\hbox{${{\text{I}}_{{{\text{photo}}}} }$} \!\mathord{\left/ {\vphantom {{{\text{I}}_{{{\text{photo}}}} } {{\text{A}} \cdot {\text{E}}}}}\right.\kern-\nulldelimiterspace} \!\lower0.7ex\hbox{${{\text{A}} \cdot {\text{E}}}$}} $$

8 $$ {\text{D }}\left( {{\text{Jones}}\,\left( {{\text{or}}} \right)\,{\text{cm}}\;{\text{Hz}}^{0.5} \;{\text{W}}^{ - 1} } \right) = { }\left( {{\raise0.7ex\hbox{${\text{R}}$} \!\mathord{\left/ {\vphantom {{\text{R}} {\left( {\sqrt {2 \cdot {\text{q}} \cdot } {\text{I}}_{{{\text{dark}}}} } \right)}}}\right.\kern-\nulldelimiterspace} \!\lower0.7ex\hbox{${\left( {\sqrt {2 \cdot {\text{q}} \cdot } {\text{I}}_{{{\text{dark}}}} } \right)}$}}} \right) $$

where, ${\text{A}}$ is the device active area $\left( {1 \times 0.1\;{\text{cm}}^{2} } \right)$, ${\text{E}}$ is the light intensity $\left( {100\;{\text{mW}}\;{\text{cm}}^{ - 2} } \right)$ and ${\text{q}}$ is the coulombic charge $\left( {1.6 \times 10^{ - 19} \;{\text{C}}} \right)$.

From the above equations, the photo current (0.6 and 0.8A), sensitivity (150 and 400%), responsivity (150 and 400 ${\text{A}}/{\text{W}}^{ - 1}$) and detectivity (1.08 × 10^12^ and 2.89 × 10^12^ Jones in respect of samples Z and Zʹ were determined. It was observed from the Figure that, the photo current got enhanced several times the dark current in case of sample Z and Zʹ respectively. This is because, when the light is illuminated on the samples, electron–hole pairs (excitons) are generated in the GaN. The charge carriers thus generated flow in the graphene through the applied bias voltage resulting in photocurrents. As mentioned earlier, due to the interfacial differences, sample Z exhibit less photo current value when compared to sample Zʹ. It is to be noted that, the presence of a SLG underneath GaN with negligible defects (${\text{I}}_{{{\text{YL}}}} /{\text{I}}_{{{\text{NBE}}}}$ from PL) and improved material quality (N-SLG) has contributed towards drastic enhancement in photodetector performances.

**Figure 8:**
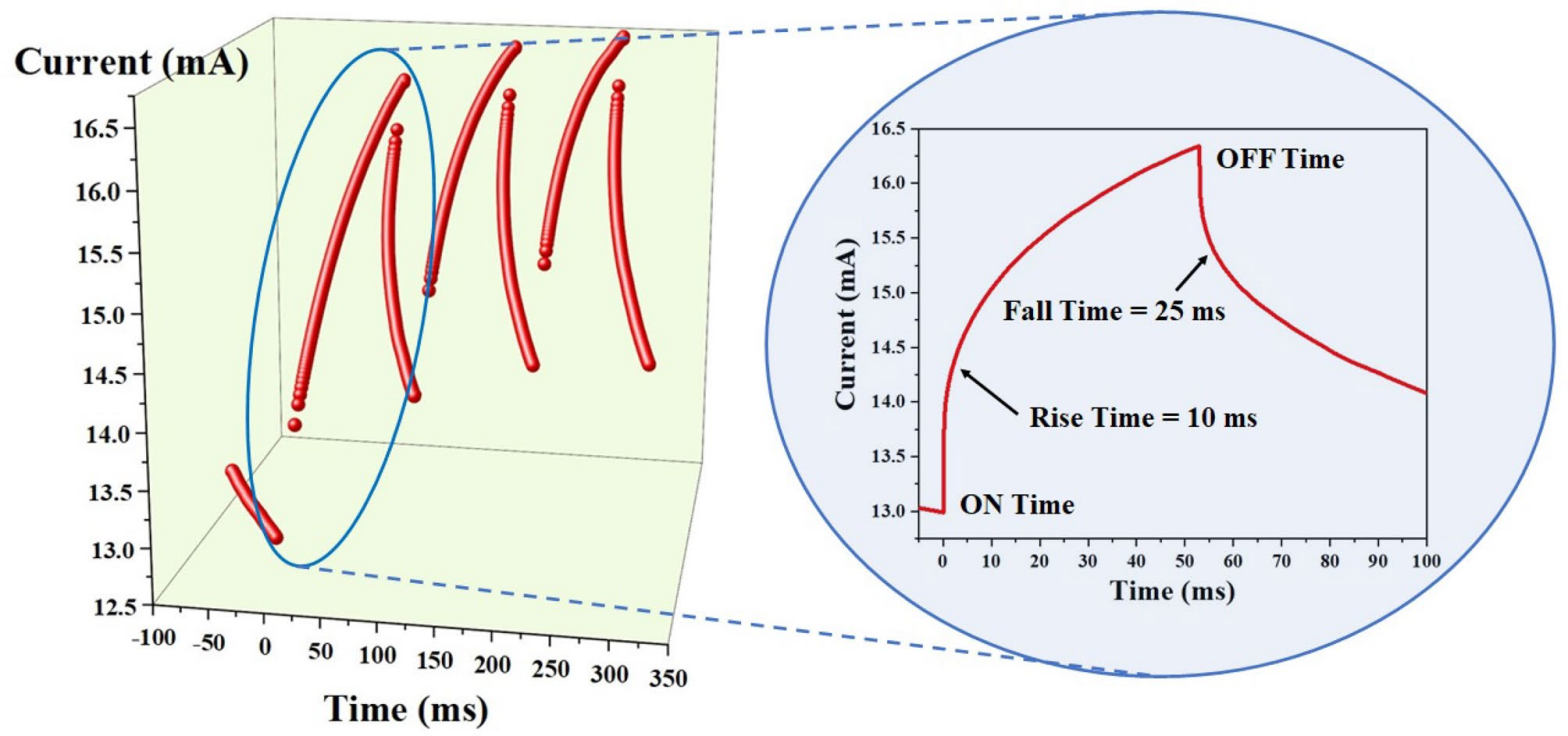
Photocurrent response of the fabricated GaN photodetector (sample Zʹ). The magnified version represents the rise time and fall time of the fabricated photodetector.

For practical opto-electronic applications, high speed switching in the photo response is a must. Since the device performance of sample Zʹ is better than that of sample Z, the switching response of the photodetector is carried out only for sample Zʹ as shown in Fig. 8. The switching response of the photodetector for the light under ON and OFF conditions can be fitted using the exponential functions based on the rise and fall time of the photodetector. The rise and fall time consist of two exponentials with different slopes (initial fast response and then a slower response). The exponential functions corresponding to rise and fall time are given as^69,70^

9 $$ {\text{I}}\left( {\text{t}} \right)_{{{\text{ON}}}} = {\text{I}}_{1} \left[ {1 - {\text{A}}_{1} {\exp}\left( {{\raise0.7ex\hbox{${ - {\text{t}}}$} \!\mathord{\left/ {\vphantom {{ - {\text{t}}} {{\uptau }_{1} }}}\right.\kern-\nulldelimiterspace} \!\lower0.7ex\hbox{${{\uptau }_{1} }$}}} \right) - {\text{A}}_{2} {\exp}\left( {{\raise0.7ex\hbox{${ - {\text{t}}}$} \!\mathord{\left/ {\vphantom {{ - {\text{t}}} {{\uptau }_{2} }}}\right.\kern-\nulldelimiterspace} \!\lower0.7ex\hbox{${{\uptau }_{2} }$}}} \right)} \right] $$

10 $$ {\text{I}}\left( {\text{t}} \right)_{{{\text{OFF}}}} = {\text{I}}_{2} \left[ {{\text{A}}_{3} {\exp}\left( {{\raise0.7ex\hbox{${ - {\text{t}}}$} \!\mathord{\left/ {\vphantom {{ - {\text{t}}} {{\uptau }_{3} }}}\right.\kern-\nulldelimiterspace} \!\lower0.7ex\hbox{${{\uptau }_{3} }$}}} \right) + {\text{A}}_{4} {\exp}\left( {{\raise0.7ex\hbox{${ - {\text{t}}}$} \!\mathord{\left/ {\vphantom {{ - {\text{t}}} {{\uptau }_{4} }}}\right.\kern-\nulldelimiterspace} \!\lower0.7ex\hbox{${{\uptau }_{4} }$}}} \right)} \right] $$

where ${\uptau }_{1}$–${\uptau }_{4}$ are the time constants and ${\text{A}}_{1}$–${\text{A}}_{4}$ are the numerical constants.

By fitting the above equations, the rise and fall time in respect of sample Zʹ is determined as 10 and 25 ms. When the light was turned ON, the photocurrent was found to rise. Similarly, when the light was turned OFF, the photocurrent was found to get dropped. In the Eqs. (9) and (10), ${\uptau }_{1}$ represents the transfer of hole from GaN to SLG, ${\uptau }_{2}$ represent the transfer of charge in GaN, ${\uptau }_{3}$ represents the lifetime of electrons in GaN before they get transferred to neighbouring graphene film and ${\uptau }_{4}$ represents the transfer of charge in GaN^70^.

The photocurrent response of the photodetector is carried out under ON/OFF condition as shown in Fig. 8. The device response remained similar even after switching the device several times. The obtained photo response is found to be faster than that of the photodetectors based on the undoped SLG reported earlier^66,71^. It is worth to note that response time is dependent on transfer rate of electrons and holes from GaN to SLG. Therefore, the nature of interface between them is crucial to the performance of the devices.

### Hall measurement study

The room temperature Hall measurements were carried out for sample Zʹ of size 10 mm^2^. It was observed that, the bulk concentration and sheet concentration of the GaN grown on N-SLG substrate was around 7.52 × 10^18^ cm^−3^ and 1.51 × 10^15^ cm^−2^ respectively. The resistivity and carrier mobility of the sample obtained was around 0.00345 Ω∙cm^−1^ and 440 cm^2^ V^−1^ s^−1^ respectively. The enhancement in electrical properties can be due to shift in the fermi level above the Dirac point of SLG as an effect of nitrogen doping, which tends to suppress the density of states near the fermi level. This action opens up the bandgap between the conduction band and valence band. In such a condition, SLG behaves as a semiconductor. As mentioned earlier, nitrogen doping regulates the property of graphene by maintaining good conductivity and carrier mobility^31^. The obtained mobility value is higher than that of the standard mobility value of GaN and also falls within the mobility range for nitrogen doped SLG as reported in literature^46,72^. The hall measurement data corroborate with I–V results.

## Conclusion

GaN epilayer was grown on N-SLG substrates using CVD technique, without the need for an interlayer between GaN and SLG for favouring the nucleation process. The results obtained using XRD revealed that GaN possess hexagonal crystal structure and GaN grown on N-SLG possess better crystalline quality. From the PL spectra, the traces observed for YL is very less. The value of ${\text{I}}_{{{\text{YL}}}} /{\text{I}}_{{{\text{NBE}}}}$ in the present case range between 0.05 to 0.15, clearly confirming that the usage of SLG has quenched the defects in GaN. The SEM, EDX and XPS results gave a detailed insight about the growth process of GaN on N-SLG substrates. SEM results clearly portrayed the morphological transformation in GaN with respect to the experimental conditions. SEM also revealed that the residual oxygen present within the CVD reactor contributed to the growth of GaN by forming GaO_x_ complex which act as nucleation sites. The results obtained from EDX and XPS emphasized the fact that in addition to residual oxygen, nitrogen and carbon also act as source of contaminations and / or doping during the growth process of GaN leading to the creation of point defects such as vacancies, interstitials and substitutional impurities and structural distortions in GaN. It is also worth to note that these actions effectively contributed towards the growth of GaN on N-SLG substrates. From the obtained electrical response, it was noticed that the estimated barrier height and ideality factor confirm that the MS interface of the photodetector is free from barrier inhomogeneities and interfacial surface states. It was observed that photo current got increased by several times in comparison to dark current. The sensitivity of the photodetector was in the range of 150 – 400%. Similarly, responsivity and detectivity of the photodetectors were estimated to be in the range of 150 – 400 ${\text{A}}/{\text{W}}^{ - 1}$ and 1.08 × 10^12^ – 2.89 × 10^12^ Jones respectively. The rise time and fall time of the photodetector was determined as 10 and 25 ms. The hall measurement values showcased high carrier mobility, sheet and bulk concentration values for GaN grown on N-SLG substrates. Hall measurement values once again confirm that, due to high content of electrons the electrical response improved drastically and resulted in high performance of photodetector.

## Experimental methods

SLG was synthesized on a copper foil using CVD system by utilizing methane (CH_4_) as the carbon precursor^73^. The growth of SLG was carried out at 1,000 °C for about 2 min by utilizing hydrogen (H_2_) as the carrier gas. During the growth process, the flow rates of CH_4_ and H_2_ were maintained at around 35 and 2 SCCM (standard cubic centimetre per minute) respectively. Post growth, the SLG was transferred onto sapphire substrate of size 1 cm^2^ (hereafter referred as SLG substrates)^74^.

The SLG substrates were then loaded into a horizontal flow CVD reactor for epitaxial growth of GaN as described elsewhere^75^. Gallium metal and liquid ammonia (NH_3_) were used as the precursors of gallium (Ga) and nitrogen (N). The growth of GaN was carried out by varying the growth time (60, 90 and 120 min) and precursor-to-substrate distance (3, 5 and 7 cm) respectively. These samples were named as X, Y, Z (in respect of growth time) and Xʹ, Yʹ and Zʹ (in respect of precursor-to-substrate distance). The growth was carried out at 900 °C by utilizing nitrogen (N_2_) as the carrier gas. During the growth, flow rate of N_2_ was maintained at 500 SCCM. The experimental conditions employed for the growth of GaN can be found in the Table 1.

**Table 1:** Experimental conditions employed for the growth of GaN on SLG substrates.

Samples	Growth conditions			
	Growth temperature (°C)	N_2_ flow rate (SCCM)	Precursor-to-substrate distance (cm)	Growth time (min)
X	900	500	3	60
Y			3	90
Z			3	120
Xʹ			3	120
Yʹ			5	120
Zʹ			7	120

The metal–semiconductor-metal (MSM) photodetectors were then fabricated on GaN using the standard procedures such as optical lithography, metallization and lift-off procedures. Gold (Au) metal of 150 nm thickness was used for metallization using sputtering system^65^.

The structural characteristics of the GaN epitaxially grown on N-SLG substrates were studied using x-ray diffractometer (XRD, PAN analytical X’Pert PRO)^35,65,75^. The surface morphologies of the GaN were investigated using scanning electron microscopy (SEM, Zeiss EVO 18). The elemental contents and maps were recorded using energy dispersive x-ray (EDX) spectroscopy, which revealed the elemental compositions in the samples^35,65,75^. The surface compositions of the samples were obtained using x-ray photoelectron spectroscopy (XPS, AXIS ULTRA)^65,75^. The photoluminescence studies were at an excitation wavelength of 244 nm using argon laser (PL, Spectra Physics)^75^. The electrical and optical performances of the fabricated photodetectors were performed using solar simulator (Newport 91160A)^75^. The mobility, bulk and sheet concentrations and resistivity of the samples were inspected using Hall measurement system (ECOPIA, HMS 5,000)^76^.

## References

[1] Hashimoto T, Wu F, Speck JS, Nakamura S (2007). A GaN bulk crystal with improved structural quality grown by the ammonothermal method. Nat. Mater..

[2] Khan A, Balakrishnan K, Katona T (2008). Ultraviolet light-emitting diodes based on group three nitrides. Nat. Photon..

[3] Qian F, Li Y, Gradecak S, Wang D, Barrelet CJ, Lieber CM (2004). Gallium nitride-based nanowire radial heterostructures for nanophotonics. Nano Lett..

[4] Dong L, Yadav SK, Ramprasad R, Alpay SP (2010). Band gap tuning in GaN through equibiaxial in-plane strains. Appl. Phys. Lett..

[5] McInnes A, Sagu JS, Mehta D, Wijayantha KGU (2019). Low-cost fabrication of tunable band gap composite indium and gallium nitrides. Sci. Rep..

[6] Gorczyca I, Suski T, Strak P, Staszczak G, Christensen NE (2017). Band gap engineering of In(Ga)N/ GaN short period superlattices. Sci. Rep..

[7] Redwing JM, Tischler MA, Flynn JS, Elhamri S, Ahoujja M, Newrock RS, Mitchel WC (1996). Two-dimensional electron gas properties of AlGaN/GaN heterostructures grown on 6H–SiC and sapphire substrates. Appl. Phys. Lett..

[8] Wong WS, Sands T, Cheung NW, Kneissl M, Bour DP, Mei P, Romano LT, Johnson NM (1999). Fabrication of thin-film InGaN light-emitting diode membranes by laser lift-off. Appl. Phys. Lett..

[9] Sumiya M, Fukuda K, Yasiro S, Honda T (2020). Influence of thin MOCVD-grown GaN layer on underlying AlN template. J. Cryst. Growth.

[10] Li T, Ren G, Yao J, Su X, Zheng S, Gao X, Xu L, Xu K (2020). Study of stress in ammonothermal non-polar and semi-polar GaN crystal grown on HVPE GaN seeds. J. Cryst. Growth.

[11] Inatomi Y, Kangawa Y (2020). Theoretical study of adatom stability on polar GaN surfaces during MBE and MOVPE. Appl. Surf. Sci..

[12] Tamura K, Kuroki Y, Yasui K, Suemitsu M, Ito T, Endou T, Nakazawa H, Narita Y, Takata M, Akahane T (2008). Growth of GaN on SiC/Si substrates using AlN buffer layer by hot-mesh CVD. Thin Solid Films.

[13] Liu L, Edgar JH (2002). Substrates for gallium nitride epitaxy. Mater. Sci. Eng. R.

[14] Zhang L, Yu J, Hao X, Wu Y, Dai Y, Shao Y, Zhang H, Tian Y (2014). Influence of stress in GaN crystals grown by HVPE on MOCVD-GaN/6H-SiC substrate. Sci. Rep..

[15] Richter E, Grunder M, Schineller B, Brunner F, Zeimer U, Netzel C, Weyers M, Trankle G (2011). GaN boules grown by high rate HVPE. Phys. Status Solidi C.

[16] Luo W, Wang X, Xiao H, Wang C, Ran J, Guo L, Li J, Liu H, Chen Y, Yang F, Li J (2008). Growth and fabrication of AlGaN/GaN HEMT based on Si (1 1 1) substrates by MOCVD. Microelectron. J..

[17] Nakamura S (1998). The roles of structural imperfections in InGaN-based blue light emitting diodes and laser diodes. Science.

[18] Molnar RJ, Singh R, Moustakas TD (1995). Blue-violet light emitting gallium nitride p-n junctions grown by electron cyclotron resonance-assisted molecular beam epitaxy. Appl. Phys. Lett..

[19] Khan MA, Chen Q, Skogman RA, Kuznia JN (1995). Violet-blue GaN homojunction light emitting diodes with rapid thermal annealed p-type layers. Appl. Phys. Lett..

[20] Cherns D (2000). The structure and optoelectronic properties of dislocations in GaN. J. Phys. Condens. Matter.

[21] Weyher JL, Ashraf H, Hageman PR (2009). Reduction of dislocation density in epitaxial GaN layers by overgrowth of defect related etch pits. Appl. Phys. Lett..

[22] Suihkonen S, Svensk O, Lang T, Lipsanen H, Odnoblyudov MA, Bougrov VE (2007). The effect of InGaN/GaN MQW hydrogen treatment and threading dislocation optimization on GaN LED efficiency. J. Cryst. Growth.

[23] Lang T, Odnoblyudov MA, Bougrov VE, Romanov AE, Suihkonen S, Sopanen M, Lipsanen H (2006). Multistep method for threading dislocation density reduction in MOCVD grown GaN epilayers. Phys. Status Solidi A.

[24] Heilmann M, Munshi AM, Sarau G, Gobelt M, Tessarek C, Fauske VT, van Helvoort ATJ, Yang J, Latzel M, Hoffmann B, Conibeer G, Weman H, Christiansen S (2016). Vertically oriented growth of GaN nanorods on Si using graphene as an atomically thin buffer layer. Nano Lett..

[25] Heilmann M, Sarau G, Gobelt M, Latzel M, Sadhujan S, Tessarek C, Christiansen S (2015). Growth of GaN micro- and nanorods on graphene-covered sapphire: enabling conductivity to semiconductor nanostructures on insulating substrates. Cryst. Growth Des..

[26] Martinez A, Fuse K, Yamashita S (2011). Mechanical exfoliation of graphene for the passive mode-locking of fiber lasers. Appl. Phys. Lett..

[27] Deokar G, Avila J, Razado-Colambo I, Codron JL, Boyaval C, Galopin E, Asensio MC, Vignaud D (2015). Towards high quality CVD graphene growth and transfer. Carbon.

[28] Stankovich S, Dikin DA, Piner RD, Kohlhaas KA, Kleinhammes A, Jia Y, Wu Y, Nguyen ST, Ruof RS (2007). Synthesis of graphene-based nanosheets via chemical reduction of exfoliated graphite oxide. Carbon.

[29] Kayali E, Mercan E, Oren EE, Buke GC (2016). Few layer graphene synthesis via SiC decomposition at low temperature and low vacuum. J. Phys. D Appl. Phys..

[30] Sanjay S, Prabakaran K, Singh S, Baskar K (2018). Catalyst-free deposition of few layer graphene on c-plane sapphire substrates by drop casting technique. J. Mater. Sci. Mater. Electron.

[31] Wang H, Maiyalagan T, Wang X (2012). Review on recent progress in nitrogen-doped graphene: synthesis, characterization, and its potential applications. ACS Catal..

[32] Balushia ZYA, Miyagi T, Lin YC, Wang K, Calderin L, Bhimanapati G, Redwing JM, Robinson JA (2015). The impact of graphene properties on GaN and AlN nucleation. Surf. Sci..

[33] Chung K, Park SI, Baek H, Chung JS, Yi GC (2012). High-quality GaN films grown on chemical vapor-deposited graphene films. NPG Asia Mater..

[34] Yadav BS, Singh S, Ganguli T, Kumar R, Major SS, Srinivasa RS (2008). Highly oriented GaN films grown on ZnO buffer layer over quartz substrates by reactive sputtering of GaAs target. Thin Solid Films.

[35] Sanjay S, Prabakaran K, Baskar K (2020). Epitaxy of gallium nitride pyramids on few layer graphene for metal-semiconductor-metal based photodetectors. Mater. Chem. Phys.

[36] Wong FR, Ali AA, Yasui K, Hashim AM (2015). Seed/catalyst-free growth of gallium-based compound materials on graphene on insulator by electrochemical deposition at room temperature. Nanoscale Res. Lett..

[37] Min JW, Bae SY, Kang WM, Park KW, Kang EK, Kim BJ, Lee DS, Lee YT (2015). Evolutionary growth of microscale single crystalline GaN on an amorphous layer by the combination of MBE and MOCVD. CrystEngComm.

[38] Finsy R (2004). On the critical radius in Ostwald ripening. Langmuir.

[39] Li T, Liu C, Zhang Z, Yu B, Dong H, Jia W, Jia Z, Yu C, Gan L, Xu B, Jiang H (2018). Understanding the growth mechanism of gan epitaxial layers on mechanically exfoliated graphite. Nanoscale Res. Lett..

[40] Maliakkal CB, Hatui N, Bapat RD, Chalke BA, Rahman AA, Bhattacharya A (2016). The mechanism of Ni-assisted GaN nanowire growth. Nano Lett..

[41] Zhao ZD, Wang B, Xu W, Zhang HR, Chen ZY, Yun GH (2015). Hydride vapor phase epitaxy of GaN on self-organized patterned graphene masks. Mater. Lett..

[42] Xing Z, Ju Z, Zhao Y, Wan J, Zhu Y, Qiang Y, Qian Y (2016). One-pot hydrothermal synthesis of Nitrogen-doped graphene as high-performance anode materials for lithium ion batteries. Sci. Rep..

[43] Gohda Y, Tsuneyuki S (2012). Structural phase transition of graphene caused by GaN epitaxy. Appl. Phys. Lett..

[44] Gruart M, Feldberg N, Gayral B, Bougerol C, Pouget S, Bellet-Amalric E, Garro N, Cros A, Okuno H, Daudin B (2019). Impact of kinetics on the growth of GaN on graphene by plasma-assisted molecular beam epitaxy. Nanotechnology.

[45] Li X, Tang T, Li M, He X (2015). Nitrogen-doped graphene films from simple photochemical doping for n-type field-effect transistors. Appl. Phys. Lett..

[46] Xu W, Lim TS, Seo HK, Min SY, Cho H, Park MH, Kim YH, Lee TW (2014). Doped graphene field-effect transistors with enhanced electron mobility and air-stability. Small.

[47] Wang X, Sun G, Routh P, Kim DH, Huang W, Chen P (2014). Heteroatom-doped graphene materials: syntheses, properties and applications. Chem. Soc. Rev..

[48] Reshchikov MA, Morkoc H (2005). Luminescence properties of defects in GaN. J. Appl. Phys..

[49] Hofmann DM, Kovalev D, Steude G, Meyer BK, Hoffmann A, Eckey L, Heitz R, Detchprom T, Amano H, Akasaki I (1995). Properties of the yellow luminescence in undoped GaN epitaxial layers. Phys. Rev. B.

[50] Calleja E, Sanchez FJ, Basak D, Sanchez-Garcıa MA, Munoz E, Izpura I, Calle F, Tijero JMG, Sanchez-Rojas JL, Beaumont B, Lorenzini P, Gibart P (1997). Yellow luminescence and related deep states in undoped GaN. Phys. Rev. B.

[51] Elsner J, Jones R, Heggie MI, Sitch PK, Haugk M, Frauenheim T, Oberg S, Briddon PR (1998). Deep acceptors trapped at threading-edge dislocations in GaN. Phys. Rev. B.

[52] Lyons JL, Janotti A, Van de Walle CG (2010). Carbon impurities and the yellow luminescence in GaN. Appl. Phys. Lett..

[53] Demchenko DO, Diallo IC, Reshchikov MA (2013). Yellow luminescence of gallium nitride generated by carbon defect complexes. Phys. Rev. Lett..

[54] Ogino T, Aoki M (1980). Mechanism of yellow luminescence in GaN. Jpn. J. Appl. Phys..

[55] Fischer S, Steude G, Hofmann DM, Kurth F, Anders F, Topf M, Meyer BK, Bertram F, Schmidt M, Christen J, Eckey L, Holst J, Hoffmann A, Mensching B, Rauschenbach B (1998). On the nature of the 3.41 eV luminescence in hexagonal GaN. J. Cryst. Growth.

[56] Calleja E, Sanchez-Garcia MA, Sanchez FJ, Calle F, Naranjo FB, Munoz E, Jahn U, Ploog K (2000). Luminescence properties and defects in GaN nanocolumns grown by molecular beam epitaxy. Phys. Rev. B.

[57] Salviati G, Albrecht M, Zanotti-Fregonara C, Armani N, Mayer M, Shreter Y, Guzzi M, Melnik YV, Vassilevski K, Dmitriev VA, Strunk HP (1999). Cathodoluminescence and transmission electron microscopy study of the influence of crystal defects on optical transitions in GaN. Phys. Stat. Sol. A.

[58] Mah KW, McGlynn E, Castro J, Lunney JG, Mosnier JP, OMahony D, Henry MO (2001). Defect luminescence of GaN grown by pulsed laser deposition. J. Cryst. Growth.

[59] Rebane YT, Shreter YG, Albrecht M (1997). Stacking faults as quantum wells for excitons in wurtzite GaN. Phys. Stat. Sol. A.

[60] Reshchikov MA, Huang D, Yun F, Visconti P, He L, Morkoc H (2003). Unusual luminescence lines in GaN. J. Appl. Phys.

[61] Reshchikov MA, Jasinski J, Liliental-Weber Z, Huang D, He L, Visconti P, Morkoc H (2003). Photoluminescence from structural defects in GaN. Phys. B.

[62] Xu SJ, Wang HJ, Cheung SH, Li Q, Dai XQ, Xie MH, Tong SY (2003). Shallow optically active structural defect in wurtzite GaN epilayers grown on stepped 4H-SiC substrates. Appl. Phys. Lett..

[63] Yang H, Li J, Jia R, Yang L, Li L (2016). Catalyst-free and selective growth of hierarchical GaN nanostructure on the graphene nanosheet. RSC Adv..

[64] Chae SJ, Kim YH, Seo TH, Duong DL, Lee SM, Park MH, Kim ES, Bae JJ, Lee SY, Jeong H, Suh EK, Yang CW, Jeong MS, Lee YH (2013). Direct growth of etch pit-free GaN crystals on few layer graphene. RSC Adv..

[65] Sanjay S, Baskar K (2018). Fabrication of Schottky barrier diodes on clump of gallium nitride nanowires grown by chemical vapour deposition. Appl. Surf. Sci..

[66] Kang S, Mandal A, Chu JH, Park JH, Kwon SY, Lee CR (2015). Ultraviolet photoconductive devices with an n-GaN nanorod-graphene hybrid structure synthesized by metal-organic chemical vapor deposition. Sci. Rep..

[67] Prakash N, Singh M, Kumar G, Barvat A, Anand K, Pal P, Singh SP, Khanna SP (2016). Ultrasensitive self-powered large area planar GaN UV-photodetector using reduced graphene oxide electrodes. Appl. Phys. Lett..

[68] Lee H, Heo K, Park J, Park Y, Noh S, Kim KS, Lee C, Hong BH, Jian J, Hong S (2012). Graphene–nanowire hybrid structures for high-performance photoconductive devices. J. Mater. Chem..

[69] Shao D, Qin L, Sawyer S (2012). High responsivity, bandpass near-UV photodetector fabricated from PVA-In_2_O_3_ nanoparticles on a GaN substrate. IEEE Photon. J..

[70] Sun Z, Liu Z, Li J, Tai GA, Lau SP, Yan F (2012). Infrared photodetectors based on CVD-grown graphene and PbS quantum dots with ultrahigh responsivity. Adv. Mater..

[71] Lin F, Chen SW, Meng J, Tse G, Fu XW, Xu FJ, Shen B, Liao ZM, Yu D (2014). Graphene/GaN diodes for ultraviolet and visible photodetectors. Appl. Phys. Lett..

[72] Meyer J, Liu R, Schaller RD, Lee HP, Bayram C (2020). Systematic study of Shockley-Read-Hall and radiative recombination in GaN on Al_2_O_3_, freestanding GaN, and GaN on Si. J. Phys. Photonics.

[73] Seethamraju S, Kumar S, Madras G, Raghavan S, Ramamurthy PC (2016). Million-fold decrease in polymer moisture permeability by a graphene monolayer. ACS Nano.

[74] Zhang G, Guell AG, Kirkman PM, Lazenby RA, Miller TS, Unwin PR (2016). Versatile polymer-free graphene transfer method and applications. ACS Appl. Mater. Interfaces.

[75] Sankaranarayanan S, Kandasamy P, Krishnan B (2019). Catalytic growth of gallium nitride nanowires on wet chemically etched substrates by chemical vapour deposition. ACS Omega.

[76] Prabakaran K, Ramesh R, Arivazhagan P, Jayasakthi M, Sanjay S, Surender S, Pradeep S, Balaji M, Baskar K (2019). Effects of indium flow rate on the structural, morphological, optical and electrical properties of InGaN layers grown by metal organic chemical vapour deposition. J. Alloys Compd..

